# The Role of Growth Directors in Controlling the Morphology of Hematite Nanorods

**DOI:** 10.1186/s11671-020-03387-w

**Published:** 2020-08-06

**Authors:** Christopher J. Allender, Jenna L. Bowen, Veronica Celorrio, Josh A. Davies-Jones, Philip R. Davies, Shaoliang Guan, Padraic O’Reilly, Meenakshisundaram Sankar

**Affiliations:** 1 CMD Ltd, Green Meadow Springs, Cardiff, CF15 7AB UK; 2 Cardiff School of Pharmacy & Pharm. Sciences, Cardiff University, Cardiff, CF10 3NB UK; 3 Diamond Light Source Ltd, Harwell Science and Innovation Campus, Oxfordshire, Didcot OX11 0DE UK; 4 Cardiff Catalysis Institute, School of Chemistry, Cardiff University, Cardiff, CF10 3AT UK; 5 Molecular Vista, 6840 Via Del Oro Suite 110, San Jose, CA 95119 USA

**Keywords:** Nanorod, Crystal growth, Aspect ratio, PIFM, Nanospindle

## Abstract

The control of the growth of hematite nanoparticles from iron chloride solutions under hydrothermal conditions in the presence of two different structure promoters has been studied using a range of both structural and spectroscopic techniques including the first report of photo induced force microscopy (PiFM) to map the topographic distribution of the structure-directing agents on the developing nanoparticles. We show that the shape of the nanoparticles can be controlled using the concentration of phosphate ions up to a limit determined to be ~6 × 10^−3^ mol. Akaganéite (β-FeOOH) is a major component of the nanoparticles formed in the absence of structure directors but only present in the very early stages (< 8 h) of particle growth when phosphate is present. The PiFM data suggest a correlation between the areas in which phosphate ions are adsorbed and areas where akaganéite persists on the surface. In contrast, goethite (α-FeOOH) is a directly observed precursor of the hematite nanorods when 1,2-diamino propane is present. The PiFM data shows goethite in the center of the developing particles consistent with a mechanism in which the iron hydroxide re-dissolves and precipitates at the nanorod ends as hematite.

## Introduction

With potential applications in analytical chemistry, catalysis, magnetic resonance imaging, and nanomedicine, the synthesis of magnetic nanoparticles and identifying strategies to control their size and morphologies are the subject of a great deal of fundamental research [1–4]. Magnetic properties facilitate both physical manipulation of particles as well as the ability to selectively heat them by magnetic hysteresis energy loss. However, the physical properties of the nanoparticles such as size, shape, and degree of crystallinity are important parameters that need to be optimized in many potential applications. To influence these properties additional “structure directing” components are often added to the synthesis procedure. Understanding the role of such additives is important if nanoparticles with improved aspect ratios and crystallinity are to be achieved. In many cases, very different additives result in similar particle growth but it is not clear whether the mechanisms by which they control growth are similar.

The precipitation of paramagnetic hematite nanorods from ferric chloride solutions is a case in point, two contrasting structure-directing additives, phosphate ions [5] and 1,2-diaminopropane [6], lead to hematite “nanorods” as the final product but since the 1,2-diaminopropane-directed rods achieve higher crystallinities and larger aspect ratios, it is possible the two processes involve different mechanisms. There has been considerable discussion of the mechanisms involved [7–10], but the picture is complicated by the range of conditions under which nanorod synthesis is performed, phosphate-directed nanorods have been synthesized at temperatures ranging from ~100 °C [11] to 210 °C [10] for example whilst the FeCl_3_:PO_4_^−^ ratio ranges from 6:1 to 40:1.

Studying the formation mechanism of such nanoparticles is also hindered by the difficulty of identifying individual components and phases at the nanoscale; transmission electron microscopy (TEM) is extremely informative but is only really effective in areas of good structural order leading to the potential to neglect amorphous regions.

The aim of this study is to explore some of the factors that influence the synthesis of hematite nanorods, in particular the concentration of phosphate ions, and to compare results using two different structural promoters. Whilst it is not possible to use identical conditions for the two directors [6], the study is the first to combine bulk and surface averaging analytic methods such as x-ray absorption spectroscopy (XAS), powder x-ray diffraction (PXRD), and x-ray photoelectron spectroscopy (XPS) with photo-induced force microscopy (PiFM) a combined surface topographic and vibrational probe, which has a lateral resolution of better than 10 nm. This combination of complementary techniques has enabled us to investigate the local environment of the nanoparticles as a function of topography at different stages of nanoparticle growth. The results show the presence and location of different intermediates during particle growth. In the case of the phosphate-directed particles, akaganéite is observed in a narrow band around the developing hematite nanoparticle early in the synthesis whereas goethite is evident in the center of the nanorods developing in the presence of 1,2-diaminopropane. Finally, PiFM data shows the presence of carbonates on both phosphate and diaminopropane-directed rods in areas also associated with the iron hydroxides and likely to be areas of nanorod growth.

## Experimental Methods

### Synthesis of Phosphate-Directed Hematite Nanorods

Hematite nanorods were prepared using the method developed by Ozaki et al. [5] in which a FeCl_3_ solution (50 ml, 4 × 10^−2^ M) containing 10 ml of between 0 and 8 × 10^−3^ M XH_2_PO_4_ (X = K or Na) is allowed to age for between 1 and 72 h at 110 °C. “Standard” synthesis conditions refers to a concentration of 5 × 10^−3^ M KH_2_PO_4_, allowed to age at 110 °C for 72 h. After separation and washing, a starting volume of 50 ml of FeCl_3_ solution typically yielded ~ 0.2 g of dried nanorods.

### Synthesis of 1,2-Diaminopropane-Directed Hematite Nanorods

FeCl_3_ (7 ml of 0.86 M solution) was added to a glass pressure reactor and stirred in an ice bath. 1,2-Diaminopropane (7 ml) was added slowly to the flask and allowed to stir in the ice bath for 15 minutes. The flask was then sealed and heated at 180 °C for 16 h. After cooling, the nanoparticles were separated by centrifugation and washed thoroughly with ethanol and water before freeze drying. Yield of product was about 0.62 g.

### Synthesis of Magnetite and Maghaemite Nanorods

Magnetite rods were prepared by freeze drying a suspension of the phosphate-directed hematite rods and placing the resulting solid in a furnace for 6 h at 350 °C in a reducing gas atmosphere (10:1 N_2_/H_2_).

### Characterization

For mechanistic studies of the growth of the nanoparticles using pXRD, XAS and Raman, the nanorods were extracted from solution by centrifugation and deposited on silica substrates without further washing. For XPS, TEM, and PiFM, the nanoparticles were isolated with a series of washing/centrifugation steps and vacuum dried onto silica, mica, and carbon-coated copper grids respectively.

For XPS the dried nanorods were pressed into conductive tape and analyzed with a Kratos Axis Ultra-DLD photoelectron spectrometer with a monochromatic Al Kα x-ray source in the “hybrid spectroscopy” mode. The analysis area was approximately 700 × 300 μm. A pass-energy of 40 eV was used for high-resolution scans and 60 eV for survey scans. CasaXPS [12] was used to analyze the spectra. Binding energies are referenced to the largest C(1 s) peak at 284.7 eV with an uncertainty of ~ 0.2 eV. Since intensities for powder samples are dependent on the surface area analyzed, which can be poorly reproducible between different powder samples, XP spectra in the figures are normalized to the point of maximum intensity.

Powder X-ray diffraction (PXRD) was performed using a PANalytical X’Pert Pro diffractometer with a monochromatic Cu Kα source (*λ* = 0.154 nm) operated at 40 kV and 40 mA. The scans were recorded over the 2*θ* range 10–80°.

X-ray absorption spectroscopy (XAS) measurements were performed on beamline B18 at Diamond Light Source [13]. Calibration of the monochromator was conducted using an iron metal foil prior to the measurements. Pellets of the different samples were collected in transmission mode at the Fe K-edge (7111 eV) simultaneously with the foil. The data were analyzed using the Athena program [14].

## Results

The nanorods generated using the two different structure-directing molecules, phosphate and 1,2-diamino propane, were examined with a range of techniques and are discussed below. The results are classified in terms of shape and crystallography and, subsequently, their spectroscopic properties.

### Shape and Crystallography

#### Transmission Electron Microscopy (TEM)

##### Phosphate-Directed Nanorods

**Fig. 1:**
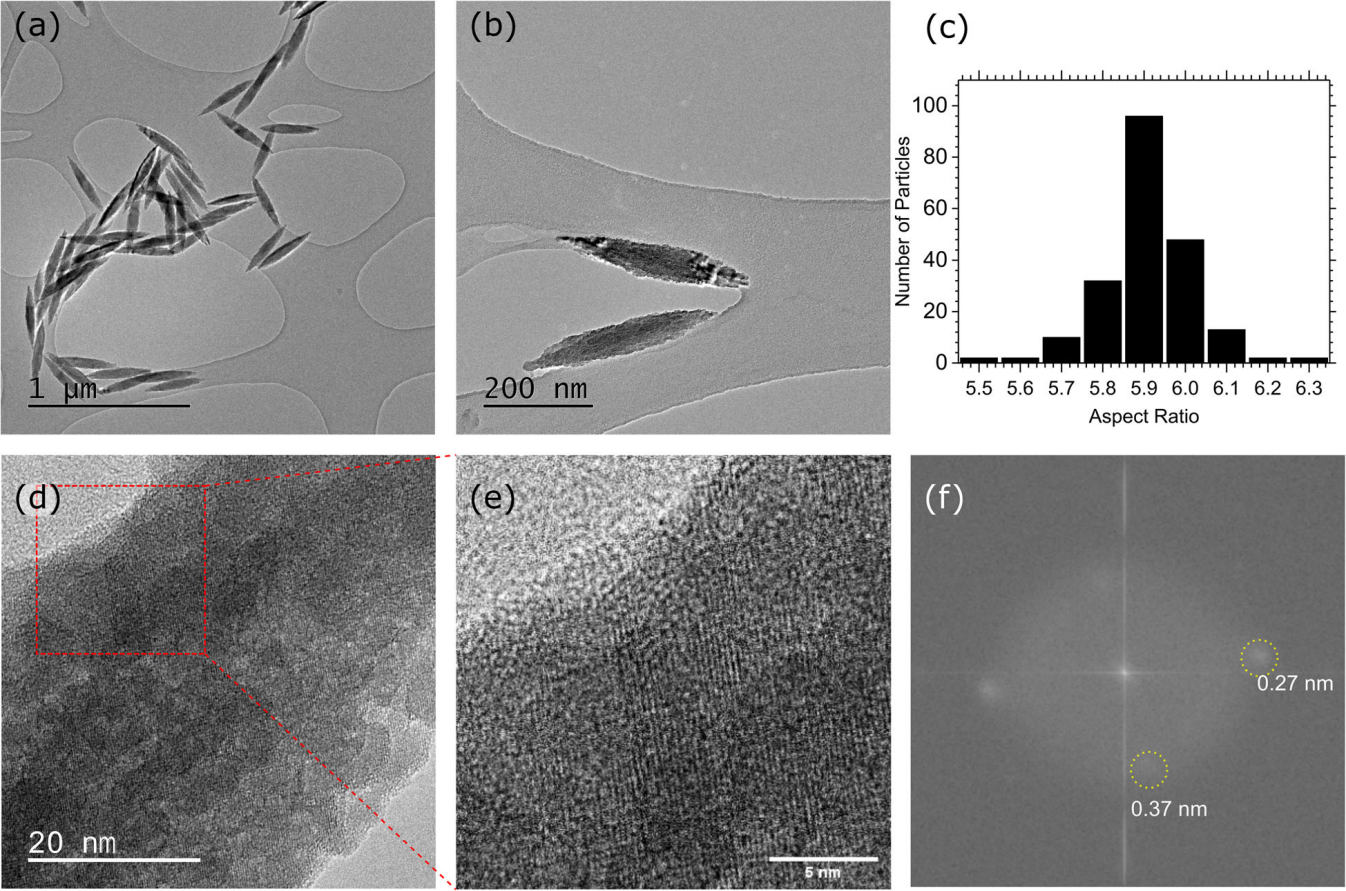
TEM studies of hematite nanorods. **a**, **b** Typical hematite nanorods synthesized in the presence of 4 × 10^−3^ M phosphate capping agent at 110 °C for 72 h. **c** The distribution of aspect ratios amongst ~ 200 hematite nanorods synthesized in the presence of 4 × 10^−3^ M phosphate capping agent at 110 °C. **d**, **e** Close up TEM micrographs of hematite nanorods showing some of the lattice fringing. **d** Fast Fourier transform of the lattice fringe showing the *d*-spacing of the hematite nanorod sample

TEM images of the phosphate-directed nanorods synthesized at a phosphate concentration of 4 × 10^−3^ mol are shown in Fig. 1. The ellipsoid shape and uneven surface of the nanorods is clearly visible in (a), and a relatively well-defined mean aspect ratio (length/width) of 5.91 was calculated from a sample of ~ 200 particles (b). A higher magnification TEM micrograph of a particle in (c) shows few signs of long-range structure with only occasional examples in the images of distinct lattice fringes.

It was not possible to determine any direction of growth along any particular crystal planes. Where lattice fringing was identified, fast Fourier transform (FFT) indicates two d-spacings at 0.27 nm, consistent with the (104) plane of hematite and in agreement with the most intense peak of the pXRD pattern, and 0.376 nm, consistent with the hematite (012) plane.

##### Effect of Phosphate Concentration on the Nanorod Aspect Ratio

**Fig. 2:**
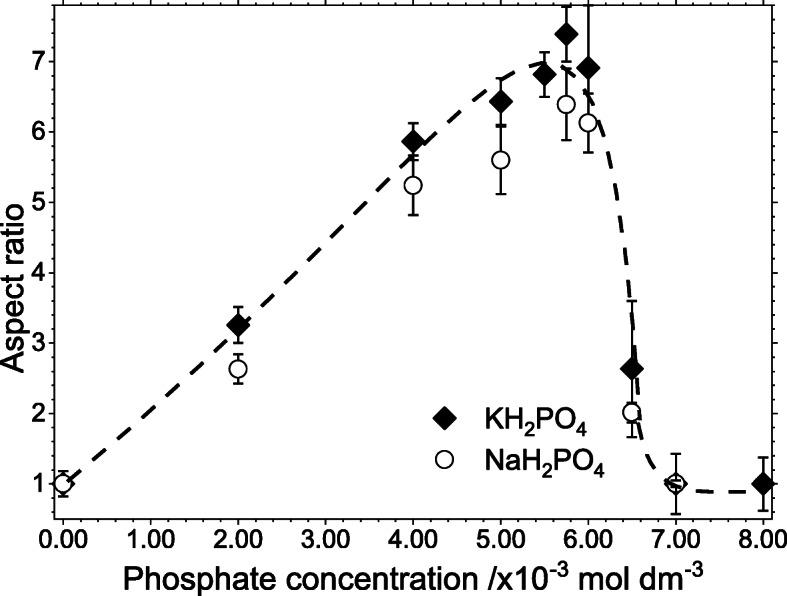
Graph of hematite nanoparticle aspect ratio as a function of potassium and sodium phosphate concentration. The figure demonstrates that the difference between the two cations is only just outside of experimental error and the concentration limits of the approach are very similar. The dashed line is drawn to guide the eye. Errors were calculated from repeated measurements of ~ 100 nanoparticles for each point

Previous work by Ma et al. [7] has shown that different quantities of the phosphate-directing agent affects the final morphologies of the nanoparticles; however, their study did not include a detailed determination of the extent to which the aspect ratio could be controlled. Using sodium and potassium phosphate concentrations between 0 and 8 × 10^−3^ M, Fig. 2 shows an elongation of the nanoparticles as the phosphate concentration is increased from 0 to ~6 × 10^−3^ M, but above 6 × 10^−3^ M, there is a precipitous drop in aspect ratio. By 7 × 10^−3^ M, there is almost no morphological control. Figure 2 also shows that whilst sodium phosphate-directed rods are always slightly shorter than the potassium phosphate-directed rods, the difference between the two cations is close to experimental error.

### 1,2-Diaminopropane-Promoted Nanorod Growth

**Fig. 3:**
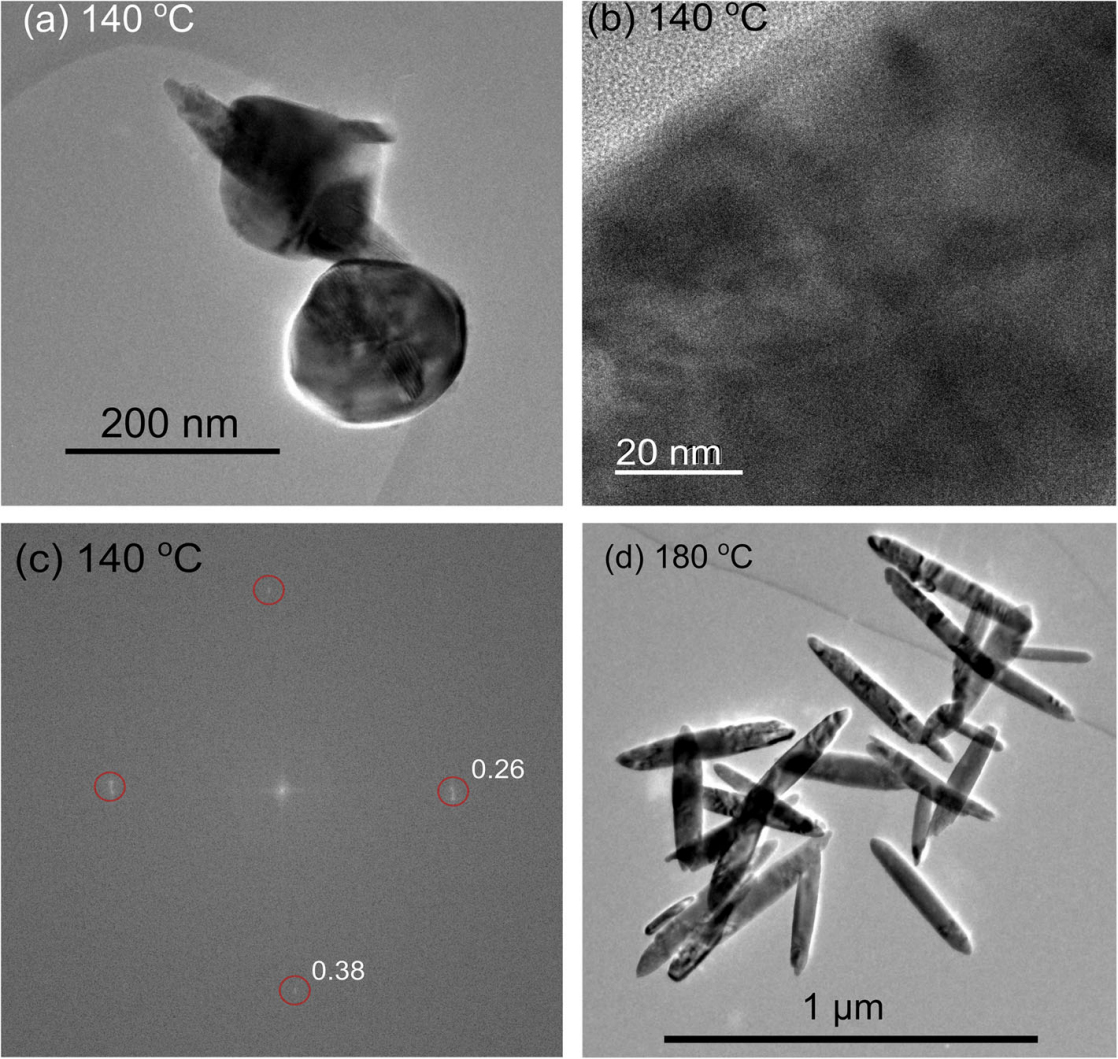
TEM of hematite nanoparticles synthesized at two different temperatures in the presence of 1,2-diaminopropane as a structure-directing additive. **a** Nanoparticles synthesized over 24 h at 140 °C. **b** Higher magnification of the developing arms of a 140 °C particle. Lattice fringes are evident suggesting good crystallinity. **c** FFT analysis of the 140 °C particle. **d** Nanoparticles synthesized over 16 h at 180 °C showing the development of nanorods with aspect ratios much larger than those obtained from the phosphate-directing agents

Figure 3 (a) and (d) show the morphology of hematite nanoparticles obtained using 1,2-diaminopropane as a structural promoter at 140 and 180 °C respectively. At the lower temperature, the nanoparticles are generally spherical in shape but do show the beginnings of the growth of rods perpendicular to the particle surface.

**Fig. 4:**
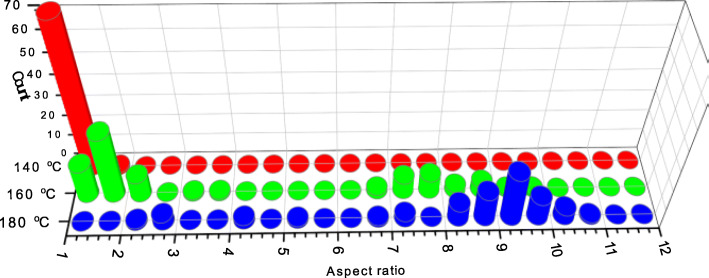
Variation of the nanorod aspect ratio as a function of aging temperature in the presence of 1,2-diamino propane as a structure-directing agent. Data was collected from ~ 75 nanorods in each sample

At higher temperatures, the proportion of the “spherical” nanoparticle intermediates decreases (see Fig. 4, for data at 160 °C) being replaced by more rod-like particles until, by 180 °C, the intermediate spherical type particles are entirely absent. A median aspect ratio of 9.25 was measured for the nanorods at this temperature. Figure 3 (a) shows 2 nanoparticles at different levels of growth at 140 °C, one with the hematite apexes protruding from the center and another without any clear protruding crystals. However, within the developing “arms” of the nanoparticle, lattice fringes can already be seen. Figure 3 (b) shows a magnified image of one of the crystal arms and (c) shows the FFT of this image.

Within the FFT, there are 2 main maxima which, like the phosphate-directed growth, correspond to the hematite (104) and (012 planes). The nanorods generated at 180 °C show higher crystallinity than the equivalent rods produced by the phosphate-directing agent. Li et al. [6] showed that changes in the concentration of 1,2 diaminopropane has much less impact on the aspect ratio of the rods than is the case with the phosphate but the temperature of aging is significant (Fig. 4). At 140 °C, the majority of particles are similar to those imaged in Fig. 3(a) with only limited anisotropy. By 160 °C, the population of spherical type particles has reduced and a substantial number of rod-like particles have developed with a median aspect ratio of ~ 8. Preparation at 180 °C gives almost exclusively nanorods with aspect ratios close to 9.25.

### Powder pXRD

**Fig. 5:**
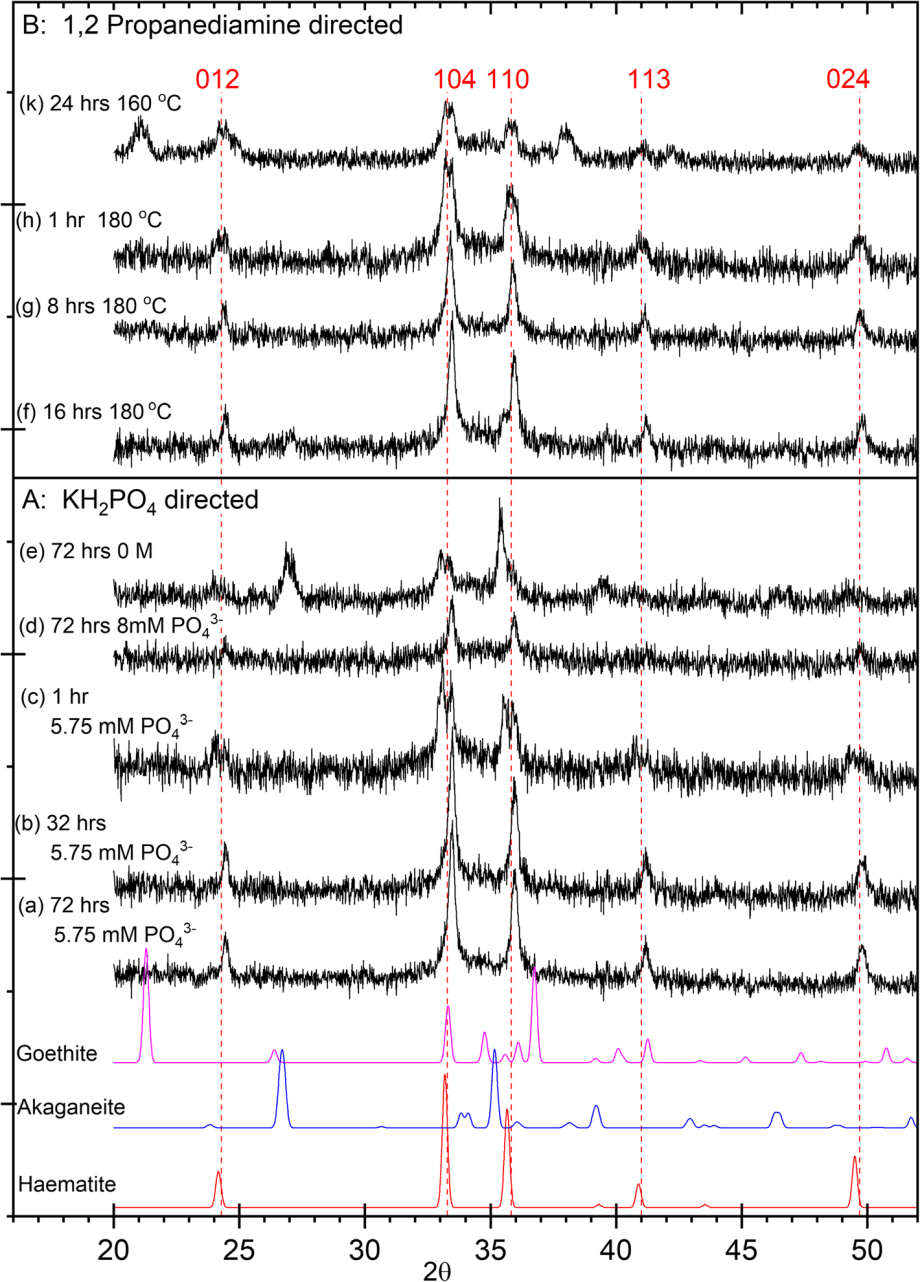
A comparison of pXRD patterns from nanorods synthesized using the two structure directors with three standard materials from the ICSD library [15–17]. **A** Phosphate-directed nanorods, prepared at 100 °C: (a)–(c) prepared with 5.75 × 10^−3^ M phosphate solution and aged for (a) 72 h; (b) 32 h; (c) 1 h; (d) prepared with 8.00 × 10^−3^ M phosphate solution and aged for 72 h; (e) prepared in the absence of phosphate ions, aged for 72 h. **B** 1,2-Diaminopropane-directed nanorods (f) aged for 16 h at 180 °C; (g) aged for 8 h at 180 °C; (h) aged for 1 h at 180 °C; (k) aged for 24 h at 160 °C

The development of the nanorods synthesized under a range of conditions for both directing agents was studied by powder pXRD (Fig. 5). The characteristic hematite [15] (012) and (104) reflections that were found in the FFT from the TEM images, together with the (110) reflection, are evident in all of the samples but most clearly in the phosphate and 1,2-diaminopropane-directed rods synthesized under standard conditions and aged for 8 h or more (Fig. 5a, b, f, g).

For the phosphate-directed rods, the quality of the hematite pattern degrades as the aging time is shortened to the extent that the rods aged for only 1 h have extensive broadening of the (110) and (104) hematite reflections and strong evidence for the presence of akaganéite [16]. The rods developed in the absence of any directing agent, but allowed to age for 16 h, give an pXRD pattern indicating that akaganéite is the dominant state (Fig. 5e). At the other end of the scale, in the presence of 8 × 10^−3^ M of phosphate, the pXRD patterns show no evidence for akaganéite with hematite the only structure present. This is despite the TEM images showing no well-defined particles.

For the diaminopropane-directed rods, reducing the aging time also degrades the hematite pattern, but even at 1 h of aging, the hematite structure is more distinct than is the case with the phosphate. The rods aged at 160 °C, however, show additional peaks characteristic of the (110), (130), and (221) lines of α-FeOOH (goethite) [17].

### Spectroscopy

#### Raman

**Fig. 6:**
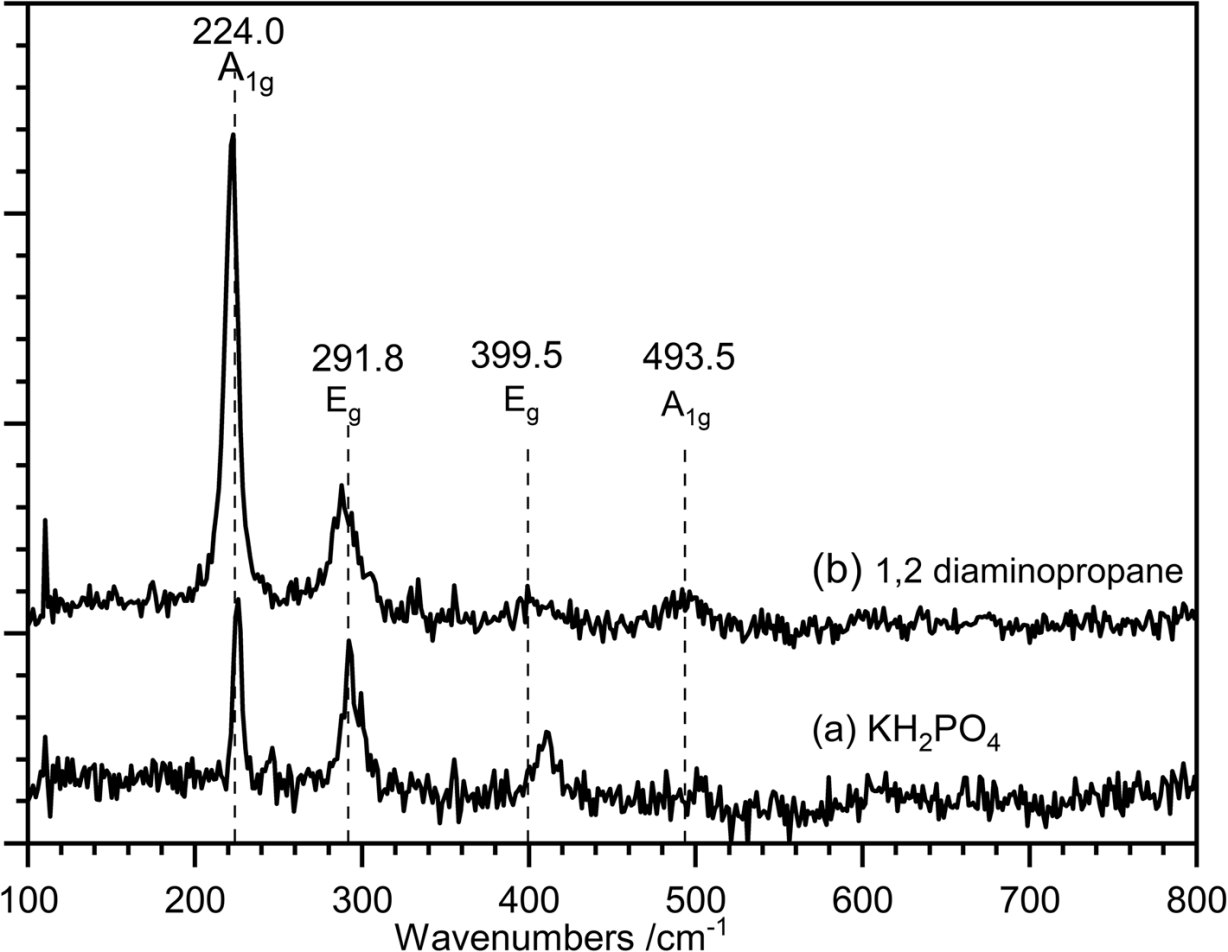
Raman spectra of hematite nanoparticles. (a) Prepared at 100 °C with 5.75 × 10^−3^ M phosphate solution and aged for 72 h; (b) 1,2-diaminopropane-directed nanorods prepared at 180 °C and aged for 16 h

Raman spectra of the hematite nanorods prepared using the two different structure-directing agents are shown in Fig. 6. The spectrum for the 1,2-diaminopropane promoted nanorods shows better signal to noise ratio than the phosphate-directed rods, but this is probably related to the slightly higher concentration of rods in the sample. Both samples show four distinct peaks at ~ 220 cm^−1^, ~ 300 cm^−1^, ~ 410 cm^−1^, and ~ 500 cm^−1^ which match well with the phonon modes reported by Jubb and Allen [18] for hematite at 229 cm^−1^ (A1g), 295 cm^−1^ and 302 cm^−1^ (Eg), 414 cm^−1^ (Eg), and 500 cm^−1^ (A1g). None of the peaks that identify maghemite (365 cm^−1^ (T2g), 511 cm^−1^ (Eg), 700 cm^−1^ (A1g)) or magnetite (310 cm^−1^ (T2g), 554 cm^−1^ (T2g), 672 cm^−1^ (A1g)) are present.

It is also noticeable that the often-reported peak [19] at 660 cm^−1^ is not present in the spectra of either of the samples. This peak has been assigned to the longitudinal optical (LO) E_u_ mode of hematite, which is IR active but expected to be Raman forbidden. Its presence in Raman spectra of hematite has been attributed to the presence of significant disorder within the lattice, and its absence here points to well-ordered crystalline structures [18]. The degree of crystallinity can also be inferred from the well-resolved peaks and relatively flat baseline suggesting the particles are predominantly crystalline although with some amorphous areas.

#### X-ray Photoelectron Spectroscopy (XPS)

**Fig. 7:**
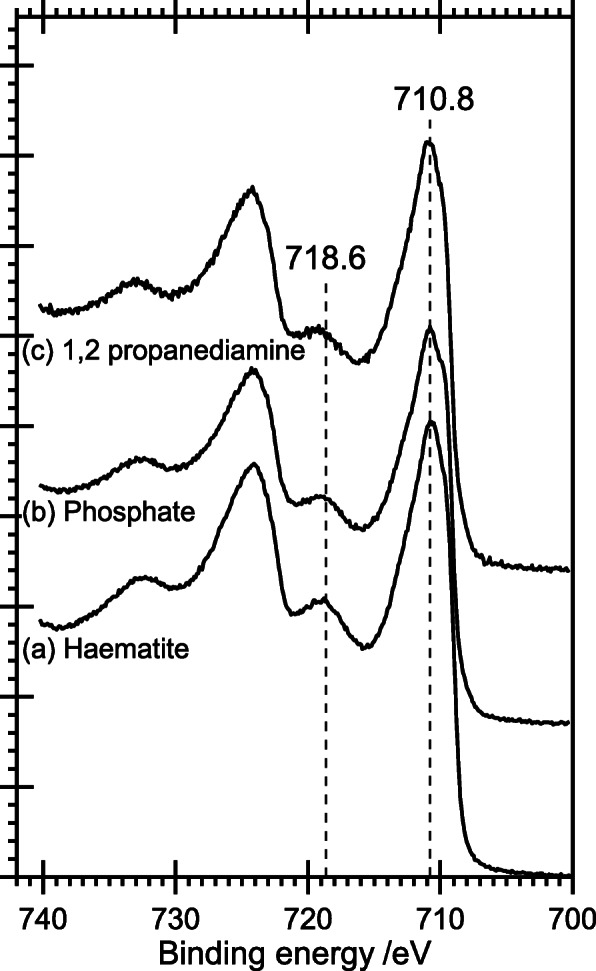
XPS: a comparison of the Fe(2p) region of the phosphate- and 1,2-diaminopropane-directed nanorods synthesized under standard conditions, with a hematite standard. (a) Hematite standard. (b) Phosphate-directed nanorods. (c) 1,2-Diaminopropane-directed rods. To allow for the different sample sizes, peaks are normalized in total area and a constant linear background subtracted from each

The Fe(2p) region of the XP spectrum is known to discriminate between different iron oxides and hydroxides. In the present case, both the “standard” phosphate and 1,2-propaneamine-directed rods show an Fe(2p_3/2_) peak at 710.7 eV, which matches literature values [20] and a reference sample of hematite (Fig. 7). The value of 710.7 eV is approximately 0.5 eV lower than expected for an iron hydroxide, and the assignment to hematite is confirmed by the clear satellite feature at 718.6 eV which is characteristic of hematite rather than the hydroxide.

#### X-ray Absorption Spectroscopic Studies (XAS)

**Fig. 8:**
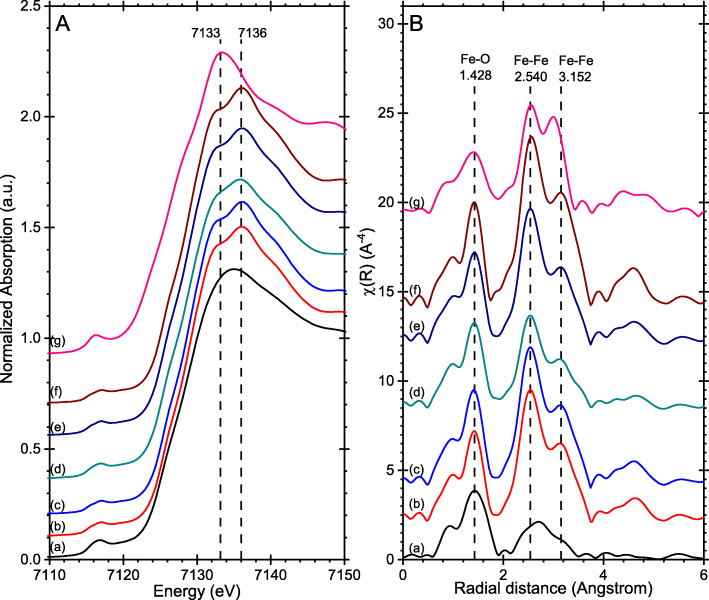
XAS spectra of hematite samples obtained under a range of conditions: **a** Normalized x-ray absorption near edge spectroscopy (XANES) spectra measured at the Fe K-edge. **b** Extended x-ray absorption fine structure (EXAFS) Fourier transforms of k^3^χ(k). (a) 6 × 10^−3^ M phosphate-directing agent aged for 1 h; (**b**) as (a) but aged for 24 h; (**c)** as (**a**) aged for 72 h; (d) no phosphate-directing agent, aged 72 h; (e) 8 × 10^−3^ M phosphate aged for 72 h; (f) 1,2-diaminopropane-directing agent aged for 16 h; (g) phosphate-directed nanorods reduced in H_2_/N_2_ at 350 °C to form magnetite rods

XAS measurements were recorded of the nanoparticles at different stages of synthesis and with varying concentrations of structure-directing agents (Fig. 8). Without the requirement for long range order, XAS provides complementary information on the iron coordination environment to PXRD. Despite the very different conditions of each sample, the changes in the XAS are quite subtle. The clearest differences are evident in the x-ray absorption near edge spectroscopy (XANES) region (Fig. 8a), where the spectrum of the phosphate-directed sample aged for just 1 h is characteristic of akagnéite [21] whereas the phosphate-directed samples aged for 24 h or longer show peak shapes characteristic of hematite [22]. These differences are also reflected in the Fe K-edge extended x-ray absorption fine structure (EXAFS) Fourier transforms (Fig. 8b) notably in the distances and relative intensities of the peaks associated to the Fe-O and Fe-Fe coordination. It is also clear from Fig. 8a, that a reduction in the concentration of phosphate reduces the hematite character of the samples and increases the akaganéite character (Fig. 8b(d)). The 1,2-diaminopropane-directed sample, which was synthesized at 180 °C, very closely matches the hematite spectrum and shows no evidence of ferric hydroxides.

#### Photo-Induced Force Microscopy (PiFM)

**Fig. 9:**
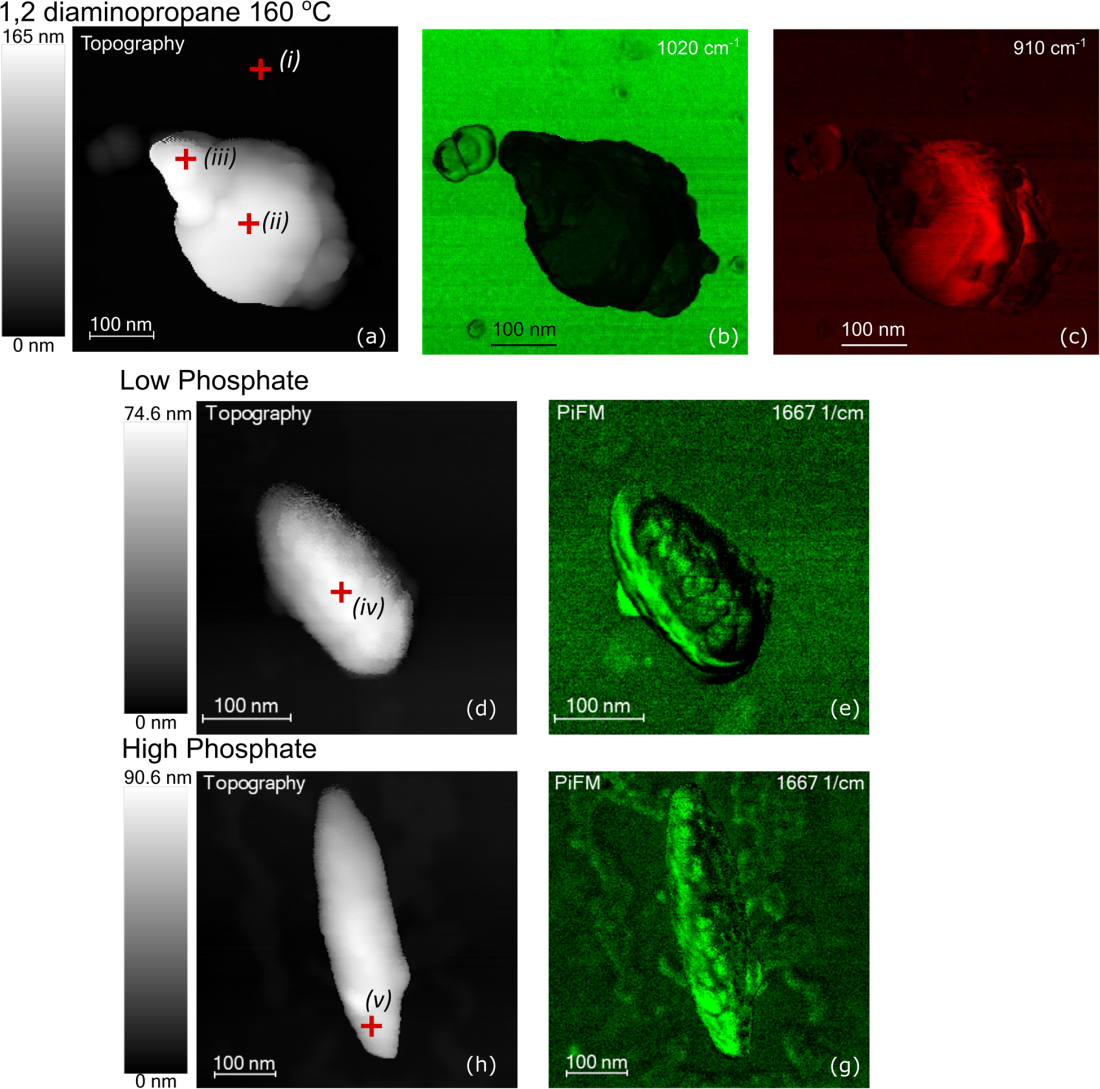
Topography (left-hand side) and PiFM intensity maps of three nanoparticles synthesized under different conditions. The frequencies at which the intensity maps were recorded are indicated on the images. **a**–**c** A 1,2-diaminopropane-directed particle synthesized at 160 °C and therefore still in the process of forming the anisotropic rods. **d**, **e** A particle synthesized in the presence of 2.5x10^−3^ M phosphate solution and aged for 48 h. **h**, **g** A particle synthesized in the presence of 6 × 10^−3^ M phosphate solution and aged for 72 h. The spectra in Fig. 10 were recorded at the points indicated by crosses on the topographic image

**Fig. 10:**
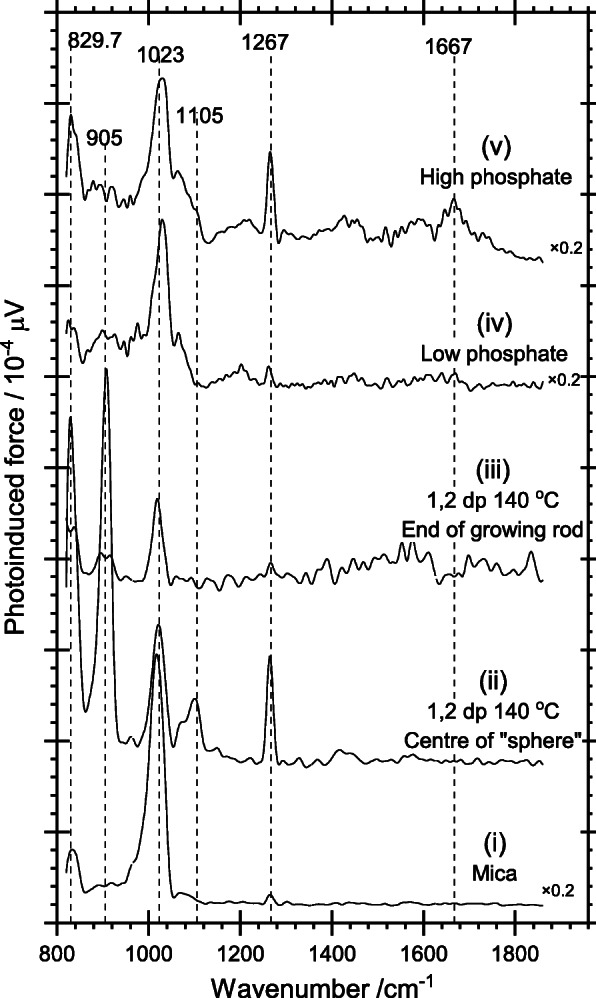
Vibrational spectra recorded using PiFM at the points indicated in the images in Fig. 9. 1,2 dp indicates -1,2-diaminopropane. (i) A spectrum of the mica support, recorded away from any particles. (ii)–(iiii)) Spectra recorded from different points on the 1,2-diaminopropane-directed nanorods. (iv),(v) Spectra recorded on selected nanorods synthesized with different concentrations of phosphate-directing agent

PiFM combines atomic force microscopy and vibrational spectroscopy in a single instrument, providing simultaneous topography and chemical signatures at the nanometer scale [23]. In the present study, this technique allowed exploration of the distribution of specific species over the surface of the nanoparticles. The topography of three rods from different synthesis conditions is shown in the images to the left-hand side of Fig. 9. Red crosses on the images show points at which the vibrational spectra in Fig. 10 were acquired whilst the images to the right of Fig. 9 show intensity maps at specific wavenumbers and the intensity of the color reflecting the strength of the specified frequency.

The background spectrum in Fig. 10 (i) was recorded at the point shown in Fig. 9, at some distance from any nanoparticle (note the × 5 reduction of Fig. 10 (i) compared to the other diaminopropane spectra). It is dominated by an intense peak at 1020 cm^−1^, which corresponds exactly with the Si-O stretch of mica. A peak at similar frequencies occurs in all the spectra recorded on the nanoparticles themselves, albeit at a weaker level. However, at these points, the tip is > 30 nm above the surface and would not detect the mica; we must conclude that the process of depositing the nanoparticles from solution leads to some redistribution of mica dust across the samples. Fortunately, mica does not have any other vibrational bands in the region 750–1850 cm^−1^ and so does not complicate the spectra any further. A medium strength peak at ~ 1265 cm^−1^ is present in some positions on both the diamino- and phosphate-directed rods but does not correspond to any previously reported band for mica, hematite, goethite, or akaganéite [24–26]. The peak is at least 100 cm^−1^ too high in frequency to be assigned to either an adsorbed phosphate or the 1,2-diaminopropane. A possible assignment is to a carbonate generated by reaction of carbon dioxide with the iron hydroxide surface as suggested by Persson et al. [27]. Alternatively, a 1265 cm^−1^ peak is characteristic of siloxane which could have been adsorbed as a contaminant.

Of most interest to this study is the strong peak at 910 cm^−1^ in (ii) assigned unambiguously to the OH deformation mode of goethite [26, 28]; mapping of this peak’s intensity across the whole sample (Fig. 9(c)) shows that the goethite is present mainly around the central spherical part of the developing particle and interestingly, completely absent from the apex.

In the phosphate-directed rods, a new feature is observed near 1667 cm^−1^, and mapping the intensity of this peak across the two different nanorods (Fig. 9e, g) shows it to be most intense around the edges of the particle growing at low phosphate concentrations but concentrated at the ends of the nanorods grown under higher phosphate concentrations. The peak can be assigned to akaganéite [28–30], and its intensity map is interesting. On the particle synthesized under low phosphate conditions, where the aspect ratio of the rods is expected to be low, akaganéite has a high concentration around the edge of the rod, but, as Fig. 10(iv) shows, is virtually undetectable in the middle of the rod. Under higher phosphate concentrations, where much better aspect ratios are expected, the akaganéite is present at the apex of the rods. This is consistent with Frandsen et al. [31] model of the development of the hematite rods in which growth occurs through the precipitation of hydroxide followed by conversion into hematite.

## Discussion

The phosphate-based preparation procedure first described by Ozaki et al. [5] reproducibly creates hematite particles with a narrow size distribution, and our results show that the aspect ratio of the particles can be precisely tuned from 1 to ~ 7.5 by increasing the FeCl_3_:PO_4_^−^ phosphate molar ratio from 100:1 to 30:1. At higher ratios, corresponding to phosphate concentrations above ~6 × 10^−3^ M, however, control of the particle shape breaks down.

The TEM images in Fig. 3 confirm previous reports that phosphate-directed hematite rods formed under the entire range of conditions we have studied lack long range crystallinity. Their appearance seems consistent with the formation mechanism proposed by Frandsen et al. [31] in which akagenéite sub-units convert into hematite after aggregation into the final “rice” shape. However, Itoh and Sugimoto [8] took a different view concluding that after an initial nucleation of akagenéite sub-units, hematite crystalizes directly from the solute fed by the dissolution of the akaganéite. In both models, the phosphate principally acts as a site blocker and as a result has an overall inhibitive effect on growth rates. Chen et al. used higher relative phosphate concentrations (FeCl_3_:PO_4_^−^ ~ 23:1 and ~ 6:1), at a much higher temperature (220 °C) [10], to successfully synthesize hematite nanorods and nanodisks. The higher temperature perhaps being necessary to counteract the overall higher phosphate concentration by reducing the equilibrium surface coverage. In Chen et al.’s work at the lower phosphate concentrations, “spindle-like particles” formed from the aggregation of “relatively stable” β-FeOOH nanorods. The outer shell of the particles subsequently crystallized to form hematite. At higher phosphate ratios, the akagenéite intermediate is not seen with hematite particles formed directly but aggregating into disks rather than rods. These observations would both appear to support the Frandsen model.

For our samples, the XPS, XANES, Raman and pXRD data show the dominant phase to be hematite with only those particles prepared at the shortest aging times, or in the absence of phosphate, showing any significant concentrations of akaganéite. The mapping of akaganéite by PiFM in Fig. 9 is interesting in this respect, showing akaganéite at the edges of the developing nanoparticles where one would expect to see growth occurring to give rod like particles, again supporting the Frandsen model. Our data makes an interesting comparison with the work of Chen et al. performed at 220 °C and higher phosphate concentrations. In both pieces of work, increasing the phosphate concentration led to more rapid conversion of the akaganéite “intermediate” into hematite, despite Sugimoto’s observation that phosphate inhibits the overall rate of nanoparticle formation. This could indicate that the whilst phosphate does block sites, it also plays a role in aiding the crystallization of the hematite from akaganéite. This is an intriguing suggestion that should be investigated in more detail.

In contrast to the phosphate-directed rod growth, varying the concentration of diaminopropane gave much less control over the nanorod anisotropy with more of an effect on the yield of rods synthesized. TEM and PIFM data support the growth mechanism suggested by Li et al. [6] for the diaminopropane-directed rods with direct evidence for the initial formation of goethite spheres followed by a recrystallization of hematite on opposing sides of the sphere until the entire particle forms a hematite rod.

## Conclusion

The extent to which the aspect ratio of hematite rods can be controlled by phosphate and 1,2-diaminopropane structure directors has been delineated. In the case of the phosphates, the aspect ratio can be tuned from between 3 and 7, whilst the diamino rods give a consistent aspect ratio of 9.8. The first use of PiFM to study the role of the structure directors on the growth of the rods has shed new light on the synthesis mechanism backing up XAS, pXRD, and XPS studies of the reactions. In particular, the data hints that the phosphate ions used to control the nanoparticle shape may also play a role in accelerating the conversion of akaganéite into hematite.

## Data Availability

The datasets generated and/or analyzed during the current study are available in the Cardiff University repository [The DOI for the dataset ‘The role of growth directors in controlling the morphology of hematite nanorods’ is 10.17035/d.2020.0112804647].

## Abbreviations

PiFM: Photo induced force microscopy
TEM: Transmission electron microscopy
XAS: X-ray absorption spectroscopy
PXRD: Powder x-ray diffraction
XPS: X-ray photoelectron spectroscopy
FFT: Fast Fourier transform
ICSD: Inorganic Crystal Structure Database
EXAFS: Extended x-ray absorption fine structure
XANES: X-ray absorption near edge spectroscopy
KESS: Knowledge Economy Skills Scholarships
ESF: European Social Fund
EPSRC: Engineering and Physical Sciences Research Council
HarwellXPS: EPSRC National Facility for Photoelectron Spectroscopy
